# Optimized dual-time-window protocols for quantitative [^18^F]flutemetamol and [^18^F]florbetaben PET studies

**DOI:** 10.1186/s13550-019-0499-4

**Published:** 2019-03-27

**Authors:** Fiona Heeman, Maqsood Yaqub, Isadora Lopes Alves, Kerstin Heurling, Johannes Berkhof, Juan Domingo Gispert, Santiago Bullich, Christopher Foley, Adriaan A. Lammertsma, on behalf of the AMYPAD Consortium

**Affiliations:** 1 Amsterdam UMC, Vrije Universiteit Amsterdam, Radiology and Nuclear Medicine, Amsterdam Neuroscience, De Boelelaan, 1117 Amsterdam, Netherlands; 2 Wallenberg Centre for Molecular and Translational Medicine and the Department of Psychiatry and Neurochemistry, University of Gothenburg, 405 30 Gothenburg, Sweden; 3 Amsterdam UMC, Vrije Universiteit Amsterdam, Epidemiology and Biostatistics, De Boelelaan, 1117 Amsterdam, Netherlands; 4 Barcelonaβeta Brain Research Center, Pasqual Maragall Foundation, Carrer de Wellington, 30, 08005 Barcelona, Spain; 5 Centro de Investigación Biomédica en Red de Bioingeniería, Biomateriales y Nanomedicina (CIBER-BBN), Av. Monforte de Lemos, 3-5. Pabellón 11. Planta 0, 28029 Madrid, Spain; 6 Universitat Pompeu Fabra, Plaça de la Mercè, 10, 08002 Barcelona, Spain; 7 Life Molecular Imaging GmbH, Tegeler Str. 7, 13353 Berlin, Germany; 8 GE Healthcare, Little Chalfont, Amersham, HP7 9NA UK

**Keywords:** Amyloid, Quantification, Simplified methods, Flutemetamol PET, Florbetaben PET, Alzheimer’s disease

## Abstract

**Background:**

A long dynamic scanning protocol may be required to accurately measure longitudinal changes in amyloid load. However, such a protocol results in a lower patient comfort and scanning efficiency compared to static scans. A compromise can be achieved by implementing dual-time-window protocols. This study aimed to optimize these protocols for quantitative [^18^F]flutemetamol and [^18^F]florbetaben studies.

**Methods:**

Rate constants for subjects across the Alzheimer’s disease spectrum (i.e., non-displaceable binding potential (BP_ND_) in the range 0.02–0.77 and 0.02–1.04 for [^18^F]flutemetamol and [^18^F]florbetaben, respectively) were established based on clinical [^18^F]flutemetamol (*N* = 6) and [^18^F]florbetaben (*N* = 20) data, and used to simulate tissue time-activity curves (TACs) of 110 min using a reference tissue and plasma input model. Next, noise was added (*N = 50)* and data points corresponding to different intervals were removed from the TACs, ranging from 0 (i.e., 90–90 = full-kinetic curve) to 80 (i.e., 10–90) minutes, creating a dual-time-window. Resulting TACs were fitted using the simplified reference tissue method (SRTM) to estimate the BP_ND_, outliers (≥ 1.5 × BP_ND_ max) were removed and the bias was assessed using the distribution volume ratio (DVR = BP_ND_ + 1). To this end, acceptability curves, which display the fraction of data below a certain bias threshold, were generated and the area under those curves were calculated.

**Results:**

[^18^F]Flutemetamol and [^18^F]florbetaben data demonstrated an increased bias in amyloid estimate for larger intervals and higher noise levels. An acceptable bias (≤ 3.1%) in DVR could be obtained with all except the 10–90 and 20–90-min intervals. Furthermore, a reduced fraction of acceptable data and most outliers were present for these two largest intervals (maximum percentage outliers 48 and 32 for [^18^F]flutemetamol and [^18^F]florbetaben, respectively).

**Conclusions:**

The length of the interval inversely correlates with the accuracy of the BP_ND_ estimates. Consequently, a dual-time-window protocol of 0–30 and 90–110 min (=maximum of 60 min interval) allows for accurate estimation of BP_ND_ values for both tracers.

[^18^F]flutemetamol: EudraCT 2007-000784-19, registered 8 February 2007, [^18^F]florbetaben: EudraCT 2006-003882-15, registered 2006.

**Electronic supplementary material:**

The online version of this article (10.1186/s13550-019-0499-4) contains supplementary material, which is available to authorized users.

## Background

Deposition of amyloid-beta (Aβ) plaques in the brain is the earliest in vivo measurable hallmark in the development of Alzheimer’s disease (AD), which is the most common type of dementia [1–3]. Therefore, visualization of Aβ deposits in vivo is essential for improving early diagnosis and monitoring treatment effects [4]. To this end, various positron emission tomography (PET) amyloid tracers have been developed [5]. Among those, fluorine-18 (18F)-labeled tracers approved by the European Medicines Agency (EMA)/Food and Drug Administration (FDA) are of special interest for clinical trials due to their relatively long half-life *t*_1/2_ = 109.8 min compared to [^11^C]PiB (Carbon-11 Pittsburgh Compound B) and commercial availability [5, 6].

In addition to visualization, amyloid PET allows for quantification of underlying physiological processes, such as the level of Aβ plaque burden [7–10]. For diagnostic purposes, a static scan acquired at pseudo-equilibrium, using a tracer-specific approved method, has been deemed sufficient in combination with visual assessment of the images. In research settings, this simplified protocol is commonly used to calculate the standardized uptake value ratio (SUVR) [5]. SUVR, however, is only a semi-quantitative parameter that is known to be affected by both scanning time window and (changes in) blood flow [11, 12]. Given this dependency, full quantification using pharmacokinetic modeling may be required to obtain higher overall sensitivity for measuring longitudinal changes (e.g., for monitoring disease progression or treatment response), especially during the early stages of the disease when amyloid is still accumulating. Pharmacokinetic modeling, however, requires a dynamic scanning protocol, which can last for up to 2 h depending on the actual tracer. These long acquisition protocols result in lower patient comfort and lesser efficient use of both scanner and tracer batch, in addition to an increased risk of motion artifacts. Dynamic data acquisition in a dual-time-window protocol (also called “coffee-break” protocol), however, can be used to reduce overall scanning time, in which data are acquired separately for early and late phases. Such a protocol provides a resting period for the patient and, when long enough, may also allow for interleaved scanning protocols, thereby optimizing tracer batch and scanner usage (i.e., costs), while maintaining a high quantitative accuracy.

So far, some studies have used a dual-time-window protocol using static acquisition of amyloid-PET data. An early scan (i.e., 0–10 min p.i.) was proposed in addition to the (standard) late static scan, as it has been reported that the early scan may provide information on metabolism and neuronal injury, possibly circumventing the need for additional [^18^F]FDG imaging [12–16]. Recently, Bullich and colleagues demonstrated that the non-displaceable binding potential (BP_ND_) obtained using a dual-time-window acquisition protocol (0–30 and 120–140 min p.i.) correlated well with BP_ND_ obtained using a full dynamic acquisition protocol of 140 min [17]. This [^18^F]florbetaben study, however, did not report details about different resting periods, nor did it assess the robustness of the dual-time-window protocol for subjects across the AD spectrum and for different noise levels (e.g., for regions of different sizes).

The purpose of the present simulation study was to define optimal dual-time-window acquisition protocols for [^18^F]florbetaben and [^18^F]flutemetamol, both in terms of patient comfort and throughput, while maintaining high quantitative accuracy. These simulations were focused on early stages of the disease, given the potential value of amyloid imaging to guide interventions aimed at secondary prevention of AD dementia.

## Methods

### Subjects and PET data

[^18^F]flutemetamol whole blood input curves, metabolite-corrected arterial plasma input curves, and time-activity curves (TACs) from 12 volumes of interest (VOIs) of three healthy controls and three probable AD subjects were obtained from Heurling et al. and Nelissen et al. [7, 18]. [^18^F]florbetaben metabolite-corrected and metabolite-uncorrected plasma input curves together with whole blood samples and TAC data from 13 VOIs of 10 healthy controls and 10 AD subjects were obtained from Becker et al. [8].

### Kinetic models for BP_ND_ estimation

**Fig. 1:**
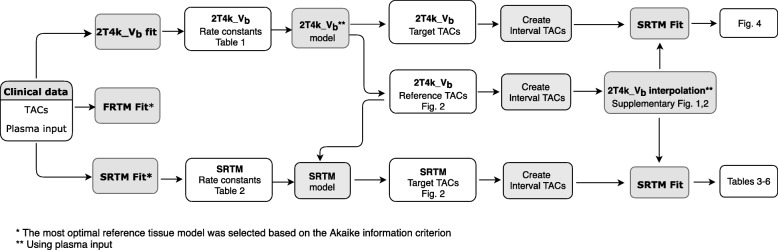
Overview of the kinetic models used during each step of the analysis

It has been shown that the reversible two-tissue compartment model (4 rate constants) with additional blood volume fraction parameter (2T4k_V_b_) is the optimal plasma input model for describing both [^18^F]flutemetamol and [^18^F]florbetaben kinetics [8, 18]. In addition, several non-invasive reference tissue-based approaches have also been used: the simplified reference tissue model (SRTM) and its basis function approach (receptor parametric mapping, RPM), the multilinear reference tissue method (MRTM), and reference Logan [19–22]. In the present study, the 2T4k_V_b_, SRTM and the full reference tissue model (FRTM [23]) were examined (Fig. 1 provides an overview of the kinetic models used during each step of the analysis). The main aim, however, was to verify the applicability of a reference tissue model approach given its applicability for large clinical trials.

### Kinetic parameters for TAC simulations

Both reported whole blood and metabolite-corrected arterial plasma input curves were used for [^18^F]flutemetamol analysis [24]. For [^18^F]florbetaben, continuous whole blood curves were generated by scaling the continuous (non-metabolite-corrected) plasma curves using discrete whole blood samples. Subsequently, all cortical and cerebellar TACs of all subjects were analyzed using the 2T4k_V_b_ model, SRTM, and FRTM [19, 23, 25]. The Akaike information ciriterion was used to determine which reference tissue method best described the kinetics of the tracer [26]. Both the optimal reference tissue model and the 2T4k_V_b_ model were then used for estimating rate constants.

Finally, from the rate constants of the cortical regions (target tissue consisting of anterior and posterior cingulate, frontal, parietal, and lateral and medialtemporal cortex) and cerebellum gray matter (reference tissue), mean and standard deviations were calculated and used to establish the range of rate constants for composite cortical and reference tissue regions. The resulting rate constants for the 2T4k_V_b_ and reference tissue model can be found in Tables 1 and 2, respectively.

**Table 1:** Pharmacokinetic parameters for simulating TACs using 2T4k_V_b_

	[^18^F]flutemetamol		[^18^F]florbetaben	
Level	BP_ND_	*k*_3_	BP_ND_	*k*_3_
BP_ND_ I	0.380	0.008	1.000	0.010
BP_ND_ II	0.635	0.013	1.500	0.015
BP_ND_ III	0.890	0.018	2.000	0.020
BP_ND_ IV	1.145	0.023	2.500	0.025
BP_ND_ V	1.400	0.028	3.000	0.030
	${\mathrm{BP}}_{\mathrm{ND}}^{\mathrm{NS}}$	k_3_	${\mathrm{BP}}_{\mathrm{ND}}^{\mathrm{NS}}$	*k*_3_
Reference	0.350	0.018	0.950	0.007

*BP*_*ND*_ binding potential of target tissue, V_b_ had a constant value of 0.05. [^18^F]flutemetamol target tissue: *K*_1_ = 0.248, *k*_2_ = 0.08, reference tissue: *K*_1_ = 0.32, *k*_2_ = 0.103. [^18^F]florbetaben target tissue: *K*_1_ = 0.226, *k*_2_ = 0.069, reference tissue: *K*_1_ = 0.25, *k*_2_ = 0.076

**Table 2:** Pharmacokinetic parameters for simulating TACs using SRTM

	[^18^F]flutemetamol	[^18^F]florbetaben
Level	BP_ND_	BP_ND_
BP_ND_ I	0.020	0.021
BP_ND_ II	0.208	0.277
BP_ND_ III	0.397	0.532
BP_ND_ IV	0.585	0.787
BP_ND_ V	0.774	1.042

*BP*_*ND*_ binding potential of target tissue, [^18^F]flutemetamol: *R*_1_ = 0.775, *k*_2_ = 0.02, [^18^F]florbetaben: *R*_1_ = 0.904, *k*_2_ = 0.03

### TAC simulations

#### Plasma input-generated TACs

Noiseless target and reference tissue TACs of 110 min duration were simulated (see Table 1 for kinetic parameters used) using the 2T4k_V_b_ model to assess the bias in BP_ND_ estimates when fitting these TACs with SRTM. The 2T4k_V_b_ model was also used to generate a reference tissue TAC for the SRTM simulations described in the next section.

#### SRTM-generated TACs

Using SRTM, tissue target TACs of 110 min duration were simulated for the range of BP_ND_ values observed clinically (50 TACs per BP_ND_, see Table 2 for kinetic parameters used), along the AD continuum. Various levels of typical PET noise were added to these target TACs only (coefficient of variation (COV) of 1, 2, and 5%, respectively) according to the variance model used by Yaqub et al., creating 50 TACs per noise level for each BP_ND_ [27]:

1 $$ {\sigma}_1^2=\alpha \bullet \mathrm{dcf}\bullet \mathrm{dcf}\ \frac{T}{L^2\ } $$

where ${\sigma}_1^2$ is the variance for each frame, calculated using the whole scanner true counts *T*, dcf is the decay correction factor, *L* is the frame length, and *α* is a proportionality constant signifying the variance level. In practice, most clinical TACs corresponded best with simulated TACs with 1 or 2% noise added, while TACs with 5% noise only corresponded with very small regions with a low BP_ND_ [7, 8].

#### Dual-time-window protocols

The “late frame” acquisition window of 90–110 min (used within Europe) was left intact, given that it constitutes the approved acquisition protocol for clinical use with visual analysis. Next, data points corresponding to the intervals in the dual-time-window protocols were removed from both the target and reference tissue TACs (creating “interval TACs”), ranging from 0 min (no interval) to 80 min (i.e., interval 10–90 min: a 0–10-min p.i. acquisition followed by a 90–110-min p.i. acquisition) in steps of 10 min. This resulted in a total of nine different protocols.

### Estimating parameters of interest

Missing data points in the reference tissue TACs, resulting from the introduction of the interval, were interpolated using the 2T4k_V_b_ model, which was used to fit the interval TACs together with a typical, tracer-specific input function, since a well-defined complete reference tissue input curve is required for SRTM to estimate the kinetic parameters of interest (*R*_1,_ BP_ND_, *k*_2_) [19]. A typical input function could be used for this purpose, based on the observed negligible between-subject variation in the tail of the curve. In future applications of this protocol, either an equivalent approach or an existing population-derived input function could be used, provided that a similar injection protocol is used. In addition, boundary values were set for all kinetic parameters (Additional file 1: Tables S1a and b) and for *k*_*3*_ of the 2T4k_V_b_ interpolation of the interval (lower boundary *k*_*3*_ = 0.005).

All TACs were fitted with SRTM, and DVR values were calculated as DVR = BP_ND_ + 1. This additional parameter was introduced as it is frequently used to express amyloid burden in other studies [28], and it better allows for expressing any bias in percentages due to its larger values.

### Evaluation of outcome parameters

Results of 2T4k_V_b_ and SRTM-generated TACs fitted with SRTM were checked for values that were physiologically not expected, here called outliers (≥ 1.5∙max simulated BP_ND_). These outliers were registered and removed from the overall dataset before further analysis. Subsequently, bias as induced by the interval was assessed for all simulated dual-time-window protocols, by calculating the bias between simulated BP_ND_ $\Big({\mathrm{BP}}_{\mathrm{ND}}^{\mathrm{sim}}$) and corresponding mean fitted BP_ND_ (${\mathrm{BP}}_{\mathrm{ND}}^{\mathrm{fit}}\Big)$:

2 $$ \mathrm{Bias}\ {\mathrm{BP}}_{\mathrm{ND}}={\mathrm{BP}}_{\mathrm{ND}}^{\mathrm{fit}}-{\mathrm{BP}}_{\mathrm{ND}}^{\mathrm{sim}} $$

Next, for each set of 50 simulated TACs at a given ${\mathrm{BP}}_{\mathrm{ND}}^{\mathrm{sim}}$ and noise level, an acceptability curve was computed by plotting the fraction of data versus a span of bias values ranging from 0 to 1 called the “absolute bias threshold.” These curves were generated for all ${\mathrm{BP}}_{\mathrm{ND}}^{\mathrm{sim}}$ values across all protocols, and the area under the curve (AUC) was calculated for all dual-time-window protocols, as a global measure of reliability. Finally, the percentage bias in the interval TAC-derived DVR was assessed by comparing it to DVR derived from the full-kinetic curve:

3 $$ \mathrm{Bias}\ {\mathrm{DVR}}_{\mathrm{ND}}\left(\%\right)=\frac{\ {\mathrm{DVR}}_{\mathrm{dual}-\mathrm{time}\ \mathrm{window}\ \mathrm{protocol}}-{\mathrm{DVR}}_{\mathrm{full}\ \mathrm{dataset}\kern0.5em }}{{\mathrm{DVR}}_{\mathrm{full}\ \mathrm{dataset}}}\bullet 100\% $$

Finally, also the percentage bias in *R*_1_ was assessed by comparing *R*_1_ derived from the various dual-time-window protocols with *R*_1_ obtained from the full-kinetic curve (in a similar way as for DVR, see Eq. 3).

## Results

### Kinetic parameters for TAC simulation

The Akaike information criterion showed that for [^18^F]flutemetamol (62.7%) and [^18^F]florbetaben (79.2%) SRTM was the preferred reference tissue method. Pharmacokinetic parameters derived from existing clinical data and used for simulating TACs based on the 2T4k_V_b_ model and SRTM are presented in Tables 1 and 2. The range of BP_ND_ values is equally spaced and corresponds to the BP_ND_ range present in the data, with BP_ND_ I being the lowest and BP_ND_ V the highest value. Of note, as described previously, BP_ND_ estimates are always different between 2T4k_V_b_ and SRTM. In the present study, additional differences are present, since the first corresponds to the sum of specific binding and a slow component of non-specific binding (in the target tissue), while in the latter a correction for all non-specific binding is made, provided that it is the same in target and reference tissues [18, 28, 29].

### TAC simulation

**Fig. 2:**
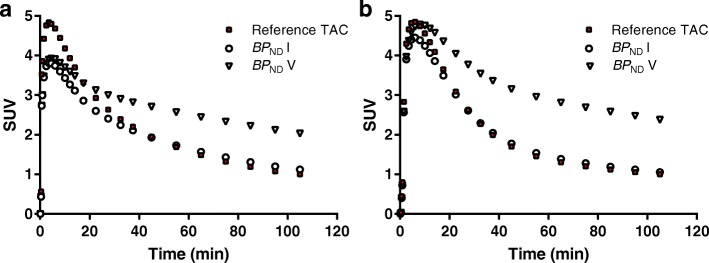
SRTM simulated target tissue TACs for the range of ${\mathrm{BP}}_{\mathrm{ND}}^{\mathrm{sim}}$ values and 2T4k_V_b_-generated reference tissue TACs for **a** [^18^F]flutemetamol and **b** [^18^F]florbetaben

**Fig. 3:**
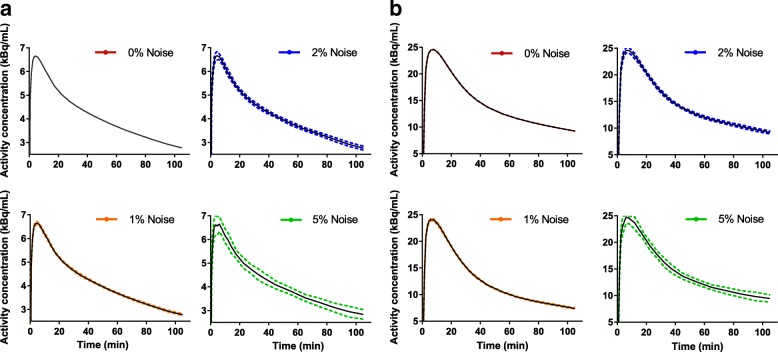
SRTM simulated TACs from a global cortical region (mean TAC value ± SD, shown solid and dashed lines, respectively) for all noise levels (COV 0–5%) for **a** [^18^F]flutemetamol and **b** [^18^F]florbetaben. All TACs were simulated using a ${\mathrm{BP}}_{\mathrm{ND}}^{\mathrm{sim}}$ III (Table 2)

Full reference and target tissue TACs, the latter covering the range of ${\mathrm{BP}}_{\mathrm{ND}}^{\mathrm{sim}}$ values, are shown in Fig. 2. Figure 3 shows the pattern in which the different noise levels were simulated for a target tissue TAC (global cortical region), resembling the shape of clinical TACs published previously [7, 8].

### Estimating parameters of interest

#### Plasma input-generated TACs

**Fig. 4:**
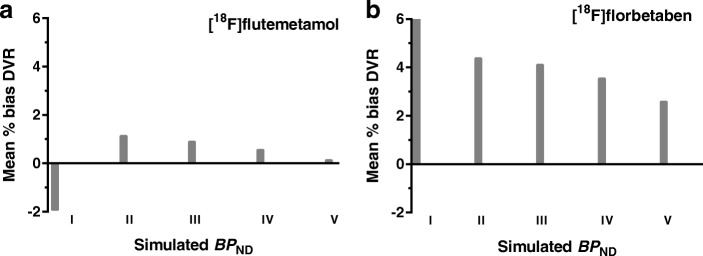
Percentage bias in SRTM-derived DVR of 2T4k_V_b_-generated TACs. **a** Bias in DVR for [^18^F]flutemetamol. **b** Bias in DVR for [^18^F]florbetaben

As can be seen in Fig. 4, SRTM-derived DVR resulted in a systematic bias when fitting the noiseless, full-kinetic curve (90–90) 2T4k_V_b_-generated TACs for both tracers. For [^18^F]flutemetamol, this bias ranged between the 0.17 and 1.95% and for [^18^F]florbetaben between the 2.62 and 6.04%. Compared to the full-kinetic curves, 2T4k_V_b_ interval TACs showed a greater bias in SRTM-derived DVR only for the 10–90 and 20–90 interval TACs (maximum bias of 3.10 and 2.25%, respectively for [^18^F]flutemetamol and maximum bias of 8.73 and 10.10%, respectively for [^18^F]florbetaben) and comparable or smaller bias for the other interval TACs.

### Evaluation of outcome parameter

#### Outliers

No outliers were present when no noise was added to the TACs, and the largest number of outliers occurred at the highest noise level for both tracers (Tables 3 and 4), full colour version see Additional file 1: Tables S2 and S3. More specifically, a positive correlation was observed between percentage of outliers and interval (*R*^2^ = 0.47, *p =* 0.04*)* and between percentage outliers and noise levels (*R*^2^ = 0.91, *p <* 0.05*)* for [^18^F]flutemetamol. For [^18^F]florbetaben, outliers were mostly present at a noise level of 5% and the number increased for higher ${\mathrm{BP}}_{\mathrm{ND}}^{\mathrm{sim}}$ values (*R*^2^ = 0.85, *p =* 0.03*)*. The percentage of [^18^F]flutemetamol outliers exceeded the 20% for the two largest intervals, while for [^18^F]florbetaben it exceeded the 20% only for the 10–90 interval.

**Table 3:** Bias in DVR and outliers as a result of noise and the dual-time-window protocol for SRTM-generated [^18^F]flutemetamol TACs

FLUT		% bias DVR			% outlier		
	Interval	1%	2%	5%	1%	2%	5%
BP_ND_ I	10–90	0.0	1.9	2.0	0.0	0.0	0.0
	20–90	6.4	6.1	2.4	0.0	0.0	0.0
	30–90	0.5	0.5	3.1	0.0	0.0	0.0
	40–90	1.5	1.6	1.6	0.0	0.0	0.0
	50–90	1.5	1.5	1.7	0.0	0.0	0.0
	60–90	1.5	1.6	1.9	0.0	0.0	0.0
	70–90	1.8	2.1	0.3	0.0	0.0	0.0
	80–90	0.8	1.3	1.3	0.0	0.0	0.0
	90–90	0.0	0.0	0.0	0.0	0.0	0.0
BP_ND_ II	10–90	1.0	6.2	8.2	0.0	2.0	12.0
	20–90	1.5	0.2	4.0	0.0	0.0	2.0
	30–90	0.1	0.2	1.3	0.0	0.0	0.0
	40–90	0.0	0.3	0.1	0.0	0.0	0.0
	50–90	0.0	0.3	0.4	0.0	0.0	0.0
	60–90	0.0	0.2	0.6	0.0	0.0	0.0
	70–90	0.0	0.1	0.6	0.0	0.0	0.0
	80–90	0.1	0.0	0.3	0.0	0.0	0.0
	90–90	0.0	0.0	0.0	0.0	0.0	0.0
BP_ND_ III	10–90	0.9	1.4	2.0	0.0	10.0	34.0
	20–90	0.1	0.5	0.6	0.0	0.0	10.0
	30–90	0.1	0.3	0.3	0.0	0.0	4.0
	40–90	0.1	0.5	0.0	0.0	0.0	4.0
	50–90	0.1	0.5	0.2	0.0	0.0	4.0
	60–90	0.1	0.5	0.1	0.0	0.0	4.0
	70–90	0.1	0.4	0.3	0.0	0.0	4.0
	80–90	0.0	0.1	0.5	0.0	0.0	2.0
	90–90	0.0	0.0	0.0	0.0	0.0	2.0
BP_ND_ IV	10–90	1.6	2.7	1.8	0.0	2.0	32.0
	20–90	0.2	0.3	0.8	0.0	0.0	12.0
	30–90	0.0	0.0	1.3	0.0	0.0	6.0
	40–90	0.0	0.1	0.6	0.0	0.0	0.0
	50–90	0.0	0.1	0.7	0.0	0.0	0.0
	60–90	0.0	0.1	0.7	0.0	0.0	0.0
	70–90	0.1	0.2	0.7	0.0	0.0	0.0
	80–90	0.0	0.1	0.9	0.0	0.0	0.0
	90–90	0.0	0.0	0.0	0.0	0.0	0.0
BP_ND_ V	10–90	1.2	0.1	6.5	2.0	14.0	48.0
	20–90	0.1	0.0	0.5	0.0	0.0	22.0
	30–90	0.0	0.6	0.8	0.0	0.0	18.0
	40–90	0.0	0.5	1.0	0.0	0.0	16.0
	50–90	0.0	0.6	0.9	0.0	0.0	14.0
	60–90	0.0	0.6	0.3	0.0	0.0	12.0
	70–90	0.0	0.4	0.5	0.0	0.0	14.0
	80–90	0.0	0.2	0.4	0.0	0.0	12.0
	90–90	0.0	0.0	0.0	0.0	0.0	12.0

Bias in simulated DVR compared to full-kinetic curve DVR and % outliers across noise levels (1–5%) for [^18^F]flutemetamol

**Table 4:** Bias in DVR and outliers as a result of noise and the dual-time-window protocol for SRTM-generated [^18^F]florbetaben TACs

FBB		% bias DVR			% outlier		
	Interval	1%	2%	5%	1%	2%	5%
BP_ND_ I	10–90	2.1	0.9	3.3	0.0	0.0	0.0
	20–90	0.9	0.2	2.3	0.0	0.0	0.0
	30–90	1.0	1.2	1.8	0.0	0.0	0.0
	40–90	0.6	1.9	0.3	0.0	0.0	0.0
	50–90	0.1	1.1	1.2	0.0	0.0	0.0
	60–90	1.0	1.0	0.4	0.0	0.0	0.0
	70–90	1.0	1.1	0.7	0.0	0.0	0.0
	80–90	0.5	0.4	0.4	0.0	0.0	0.0
	90–90	0.0	0.0	0.0	0.0	0.0	0.0
BP_ND_ II	10–90	0.1	3.4	9.1	0.0	0.0	0.0
	20–90	0.1	0.1	5.0	0.0	0.0	0.0
	30–90	0.1	0.4	0.2	0.0	0.0	0.0
	40–90	0.0	0.2	0.1	0.0	0.0	0.0
	50–90	0.0	0.2	0.3	0.0	0.0	0.0
	60–90	0.1	0.1	0.3	0.0	0.0	0.0
	70–90	0.0	0.1	0.3	0.0	0.0	0.0
	80–90	0.0	0.0	0.2	0.0	0.0	0.0
	90–90	0.0	0.0	0.0	0.0	0.0	0.0
BP_ND_ III	10–90	0.2	0.7	3.5	0.0	2.0	16
	20–90	0.2	0.2	1.8	0.0	0.0	0.0
	30–90	0.3	0.5	0.6	0.0	0.0	0.0
	40–90	0.2	0.4	0.7	0.0	0.0	0.0
	50–90	0.1	0.4	0.7	0.0	0.0	0.0
	60–90	0.1	0.4	0.8	0.0	0.0	0.0
	70–90	0.0	0.3	0.5	0.0	0.0	0.0
	80–90	0.0	0.1	0.3	0.0	0.0	0.0
	90–90	0.0	0.0	0.0	0.0	0.0	0.0
BP_ND_ IV	10–90	0.5	0.8	4.0	0.0	0.0	20.0
	20–90	0.0	0.2	1.3	0.0	0.0	0.0
	30–90	0.2	0.3	0.1	0.0	0.0	0.0
	40–90	0.0	0.2	0.2	0.0	0.0	0.0
	50–90	0.0	0.1	0.3	0.0	0.0	0.0
	60–90	0.0	0.1	0.3	0.0	0.0	0.0
	70–90	0.1	0.1	0.3	0.0	0.0	0.0
	80–90	0.0	0.0	0.5	0.0	0.0	0.0
	90–90	0.0	0.0	0.0	0.0	0.0	0.0
BP_ND_ V	10–90	0.4	1.4	2.7	0.0	0.0	32
	20–90	0.3	0.5	0.9	0.0	0.0	10
	30–90	0.3	0.8	0.7	0.0	0.0	2.0
	40–90	0.0	0.6	0.4	0.0	0.0	0.0
	50–90	0.0	0.6	0.8	0.0	0.0	2.0
	60–90	0.0	0.6	0.6	0.0	0.0	2.0
	70–90	0.0	0.4	0.2	0.0	0.0	2.0
	80–90	0.0	0.2	0.0	0.0	0.0	2.0
	90–90	0.0	0.0	0.0	0.0	0.0	0.0

Bias in simulated DVR compared to full-kinetic curve DVR and % outliers across noise levels (1–5%) for [^18^F]florbetaben

**Table 5:** Absolute bias in BP_ND_ and AUC as a result of noise and the dual-time-window protocol for SRTM-generated [^18^F]flutemetamol TACs

FLUT		Absolute mean bias BP_ND_ (SD)				AUC			
	Interval	0%	1%	2%	5%	0%	1%	2%	5%
BP_ND_ I	10–90	0.001 (0.000)	0.017 (0.058)	0.042 (0.079)	0.093 (0.154)	0.993	0.971	0.943	0.894
	20–90	0.001 (0.000)	0.084 (0.283)	0.086 (0.276)	0.098 (0.211)	0.993	0.908	0.902	0.887
	30–90	0.001 (0.000)	0.012 (0.079)	0.017 (0.074)	0.105 (0.222)	0.993	0.969	0.961	0.878
	40–90	0.001 (0.000)	0.002 (0.011)	0.006 (0.022)	0.055 (0.113)	0.993	0.986	0.977	0.929
	50–90	0.000 (0.000)	0.002 (0.011)	0.007 (0.025)	0.053 (0.116)	0.993	0.986	0.976	0.929
	60–90	0.000 (0.000)	0.002 (0.029)	0.006 (0.045)	0.051 (0.115)	0.993	0.981	0.974	0.932
	70–90	0.000 (0.000)	0.036 (0.119)	0.000 (0.020)	0.075 (0.147)	0.993	0.948	0.978	0.909
	80–90	0.000 (0.000)	0.026 (0.174)	0.035 (0.181)	0.058 (0.133)	1.000	0.955	0.946	0.923
	90–90	0.000 (0.000)	0.018 (0.162)	0.022 (0.162)	0.072 (0.202)	1.000	0.962	0.958	0.910
BP_ND_ II	10–90	0.002 (0.000)	0.014 (0.046)	0.081 (0.217)	0.128 (0.271)	0.993	0.970	0.895	0.838
	20–90	0.001 (0.000)	0.021 (0.135)	0.009 (0.040)	0.076 (0.200)	0.993	0.963	0.965	0.881
	30–90	0.001 (0.000)	0.002 (0.015)	0.003 (0.030)	0.043 (0.159)	0.993	0.982	0.971	0.906
	40–90	0.000 (0.000)	0.002 (0.015)	0.003 (0.029)	0.029 (0.112)	0.993	0.982	0.971	0.919
	50–90	0.000 (0.000)	0.002 (0.015)	0.002 (0.029)	0.022 (0.097)	0.993	0.982	0.972	0.923
	60–90	0.000 (0.000)	0.003 (0.014)	0.004 (0.029)	0.020 (0.095)	0.993	0.983	0.972	0.922
	70–90	0.000 (0.000)	0.002 (0.014)	0.005 (0.029)	0.020 (0.096)	0.993	0.984	0.972	0.924
	80–90	0.000 (0.000)	0.002 (0.013)	0.007 (0.027)	0.023 (0.098)	1.000	0.985	0.973	0.924
	90–90	0.000 (0.000)	0.003 (0.013)	0.006 (0.024)	0.027 (0.098)	1.000	0.985	0.975	0.925
BP_ND_ III	10–90	0.003 (0.000)	0.014 (0.063)	0.031 (0.099)	0.023 (0.128)	0.993	0.956	0.924	0.898
	20–90	0.002 (0.000)	0.001 (0.026)	0.019 (0.080)	0.060 (0.145)	0.993	0.975	0.946	0.876
	30–90	0.001 (0.000)	0.001 (0.023)	0.007 (0.054)	0.056 (0.158)	0.993	0.977	0.956	0.881
	40–90	0.001 (0.000)	0.001 (0.023)	0.004 (0.049)	0.051 (0.146)	0.993	0.978	0.958	0.885
	50–90	0.001 (0.000)	0.001 (0.023)	0.005 (0.048)	0.049 (0.147)	0.993	0.978	0.959	0.886
	60–90	0.001 (0.000)	0.001 (0.022)	0.005 (0.048)	0.050 (0.147)	0.993	0.978	0.959	0.887
	70–90	0.001 (0.000)	0.000 (0.021)	0.006 (0.047)	0.047 (0.144)	0.993	0.979	0.960	0.888
	80–90	0.000 (0.000)	0.001 (0.022)	0.010 (0.046)	0.059 (0.164)	1.000	0.979	0.961	0.883
	90–90	0.000 (0.000)	0.001 (0.021)	0.012 (0.043)	0.051 (0.142)	1.000	0.979	0.963	0.894
BP_ND_ IV	10–90	0.005 (0.000)	0.034 (0.082)	0.041 (0.147)	0.027 (0.192)	0.993	0.932	0.892	0.854
	20–90	0.003 (0.000)	0.012 (0.038)	0.004 (0.067)	0.043 (0.183)	0.993	0.963	0.942	0.859
	30–90	0.002 (0.000)	0.008 (0.032)	0.002 (0.055)	0.036 (0.168)	0.993	0.968	0.949	0.866
	40–90	0.001 (0.000)	0.008 (0.030)	0.003 (0.054)	0.048 (0.173)	0.993	0.970	0.950	0.858
	50–90	0.001 (0.000)	0.007 (0.031)	0.003 (0.054)	0.045 (0.174)	0.993	0.970	0.949	0.858
	60–90	0.001 (0.000)	0.008 (0.030)	0.003 (0.053)	0.046 (0.169)	0.993	0.969	0.951	0.863
	70–90	0.001 (0.000)	0.007 (0.031)	0.004 (0.051)	0.045 (0.165)	0.993	0.969	0.953	0.868
	80–90	0.000 (0.000)	0.008 (0.030)	0.000 (0.050)	0.043 (0.163)	1.000	0.969	0.954	0.867
	90–90	0.000 (0.000)	0.008 (0.028)	0.001 (0.045)	0.057 (0.179)	1.000	0.970	0.959	0.861
BP_ND_ V	10–90	0.006 (0.000)	0.023 (0.082)	0.005 (0.145)	0.117 (0.111)	0.988	0.929	0.885	0.852
	20–90	0.004 (0.000)	0.001 (0.042)	0.004 (0.090)	0.011 (0.179)	0.993	0.960	0.925	0.850
	30–90	0.002 (0.000)	0.000 (0.037)	0.014 (0.074)	0.015 (0.179)	0.993	0.965	0.938	0.851
	40–90	0.001 (0.000)	0.001 (0.034)	0.014 (0.072)	0.019 (0.168)	0.993	0.968	0.940	0.853
	50–90	0.001 (0.000)	0.001 (0.034)	0.015 (0.071)	0.018 (0.164)	0.993	0.968	0.940	0.855
	60–90	0.001 (0.000)	0.001 (0.034)	0.015 (0.071)	0.008 (0.173)	0.993	0.968	0.940	0.849
	70–90	0.001 (0.000)	0.001 (0.033)	0.012 (0.072)	0.010 (0.163)	0.993	0.968	0.939	0.856
	80–90	0.000 (0.000)	0.001 (0.031)	0.008 (0.067)	0.006 (0.172)	1.000	0.969	0.943	0.849
	90–90	0.000 (0.000)	0.001 (0.029)	0.004 (0.061)	0.002 (0.150)	1.000	0.971	0.949	0.867

Absolute bias in ${\mathrm{BP}}_{\mathrm{ND}}^{\mathrm{sim}}$ and the area under the curve (AUC) for the acceptability curves across all noise levels (0–5%) for [^18^F]flutemetamol

#### SRTM-generated [^18^F]flutemetamol interval TACs

**Fig. 5:**
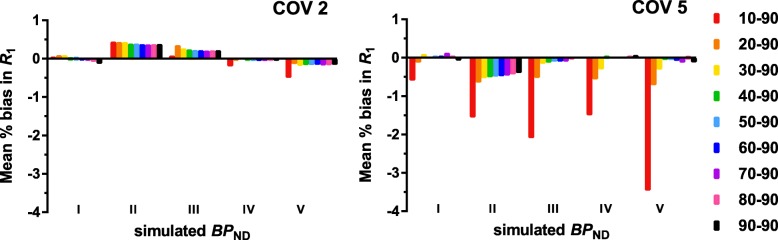
[^18^F]flutemetamol: percentage bias in SRTM-derived *R*_1_ across the range of ${\mathrm{BP}}_{\mathrm{ND}}^{\mathrm{sim}}$ values for two noise levels

The interpolated TACs can be found in Additional file 1: Figure S1. Table 5 (full color version, see Additional file 1: Table S4) shows, as expected, increasing absolute bias in ${\mathrm{BP}}_{\mathrm{ND}}^{\mathrm{fit}}$,with increasing noise levels (*R*^2^ = 0.98, *p =* 0.01) and longer intervals (*R*^2^ = 0.51, *p =* 0.03*)* (maximum bias of 0.128 for the 10–90 ${\mathrm{BP}}_{\mathrm{ND}}^{\mathrm{sim}}$ II interval, at 5% noise). In addition, there was a trend towards a negative correlation between absolute bias and ${\mathrm{BP}}_{\mathrm{ND}}^{\mathrm{sim}}$ (*R*^2^ = 0.74, *p =* 0.06*)*. The AUC values calculated from the acceptability curves show a trend of smaller (poorer) AUC values at higher noise levels (*R*^2^ = 1.00, *p <* 0.001) and larger intervals (*R*^2^ = 0.64, *p =* 0.01). As expected, the full-kinetic curve provided the highest AUC except for the lowest ${\mathrm{BP}}_{\mathrm{ND}}^{\mathrm{sim}}$. Furthermore, the 10–90 and 20–90-min intervals result in a bias in DVR of maximal 6.4%, while all other intervals showed a bias in DVR of maximum 1.6% for noise levels of up to 2%. For higher noise levels corresponding to very small regions (5%), bias in DVR was a maximum of 8.2 and 3.1%, for the 10–90 and 20–90 intervals (Table 3). Finally, Fig. 5 shows the percentage bias in *R*_1_, which was only larger than 1% for the largest interval. The bias in *R*_1_ estimates also increased with increasing noise level (*R*^2^ = 0.99, *p =* 0.004, COV2 ranging from 0.04 to − 0.497 and COV5 0.034 to − 3.462).

### SRTM-generated [^18^F]florbetaben interval TACs

**Fig. 6:**
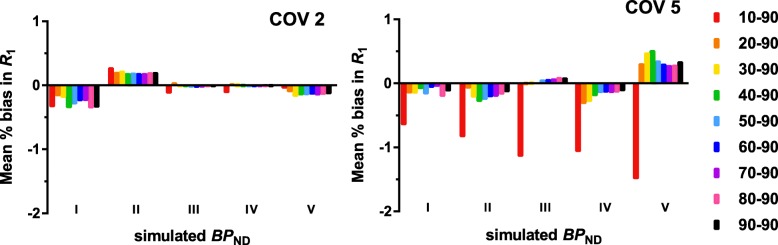
[^18^F]florbetaben: percentage bias in SRTM-derived *R*_1_ across the range of ${\mathrm{BP}}_{\mathrm{ND}}^{\mathrm{sim}}$ values for two noise levels

The interpolated TACs can be found in Additional file 1: Figure S2. Table 6 (full color version, see Additional file 1: Table S5) shows an increasing absolute bias for longer intervals (*R*^2^ = 0.48, *p =* 0.04*)* as well as for higher noise levels (*R*^2^ = 0.96, *p =* 0.02, maximum bias 1.31 for the 10–90 ${\mathrm{BP}}_{\mathrm{ND}}^{\mathrm{sim}}$ II interval, at 5%). This positive correlation was also supported by the AUC values, where lower (poorer) values were correlated with larger intervals (*R*^2^ = 0.74, *p =* 0.002*)*, higher ${\mathrm{BP}}_{\mathrm{ND}}^{\mathrm{sim}}$ values (*R*^2^ = 0.98, *p =* 0.001*)*, and higher levels of noise (*R*^2^ = 1.0, *p <* 0.001*)*. As expected, the full-kinetic curve provided the highest AUC except for the lowest ${\mathrm{BP}}_{\mathrm{ND}}^{\mathrm{sim}}$. Furthermore, the 10–90 interval showed a bias in DVR of 3.4%, all other intervals showed a bias of ≤ 1.9% for noise levels up to 2%. For higher noise levels corresponding to very small regions (5%), bias in DVR was a maximum of 9.1% for the 10–90 interval, 5.0% for the 20–90 interval, and 1.8% for all others (Table 4). The *R*_1_ bias plots (Fig. 6) show an increase in bias at higher noise levels (*R*^2^ = 0.99, *p =* 0.005, COV2 ranging from 0.001 to − 0.359 and COV5 0.003 to − 1.497).

**Table 6:** Absolute bias in BP_ND_ and AUC as a result of noise and the dual-time-window protocol for SRTM-generated [^18^F]florbetaben TACs

FBB		Absolute mean bias BP_ND_ (SD)				AUC			
	Interval	0%	1%	2%	5%	0%	1%	2%	5%
BP_ND_ I	10–90	0.000 (0.000)	0.012 (0.026)	0.021 (0.043)	0.059 (0.080)	0.993	0.979	0.966	0.929
	20–90	0.000 (0.000)	0.000 (0.011)	0.013 (0.040)	0.048 (0.076)	0.993	0.986	0.971	0.937
	30–90	0.000 (0.000)	0.000 (0.008)	0.001 (0.028)	0.006 (0.046)	0.993	0.988	0.979	0.968
	40–90	0.000 (0.000)	0.003 (0.032)	0.008 (0.014)	0.021 (0.063)	0.993	0.978	0.981	0.956
	50–90	0.000 (0.000)	0.009 (0.012)	0.000 (0.037)	0.012 (0.056)	0.993	0.983	0.978	0.962
	60–90	0.000 (0.000)	0.000 (0.007)	0.001 (0.015)	0.029 (0.063)	0.993	0.989	0.983	0.956
	70–90	0.000 (0.000)	0.001 (0.006)	0.000 (0.013)	0.032 (0.069)	0.993	0.990	0.984	0.953
	80–90	0.000 (0.000)	0.005 (0.036)	0.007 (0.066)	0.029 (0.081)	1.000	0.978	0.963	0.948
	90–90	0.000 (0.000)	0.010 (0.011)	0.011 (0.064)	0.024 (0.067)	1.000	0.983	0.962	0.956
BP_ND_ II	10–90	0.001 (0.000)	0.003 (0.013)	0.045 (0.167)	0.131 (0.307)	0.993	0.984	0.935	0.861
	20–90	0.001 (0.000)	0.000 (0.012)	0.000 (0.023)	0.079 (0.207)	0.993	0.985	0.976	0.891
	30–90	0.002 (0.000)	0.000 (0.012)	0.003 (0.023)	0.016 (0.063)	0.993	0.985	0.975	0.946
	40–90	0.000 (0.000)	0.001 (0.012)	0.001 (0.023)	0.012 (0.062)	0.993	0.985	0.976	0.946
	50–90	0.000 (0.000)	0.001 (0.011)	0.001 (0.022)	0.010 (0.060)	0.993	0.986	0.977	0.948
	60–90	0.000 (0.000)	0.002 (0.010)	0.000 (0.021)	0.009 (0.056)	0.993	0.987	0.978	0.948
	70–90	0.000 (0.000)	0.001 (0.009)	0.001 (0.020)	0.010 (0.055)	0.993	0.988	0.979	0.950
	80–90	0.000 (0.000)	0.001 (0.007)	0.002 (0.017)	0.011 (0.053)	1.000	0.989	0.981	0.954
	90–90	0.000 (0.000)	0.001 (0.008)	0.002 (0.015)	0.014 (0.053)	1.000	0.989	0.983	0.954
BP_ND_ III	10–90	0.002 (0.000)	0.002 (0.022)	0.014 (0.066)	0.084 (0.159)	0.993	0.978	0.955	0.875
	20–90	0.002 (0.000)	0.003 (0.016)	0.000 (0.041)	0.058 (0.117)	0.993	0.982	0.964	0.898
	30–90	0.003 (0.000)	0.004 (0.016)	0.004 (0.035)	0.038 (0.112)	0.993	0.982	0.966	0.910
	40–90	0.001 (0.000)	0.002 (0.016)	0.003 (0.036)	0.041 (0.116)	0.993	0.982	0.966	0.909
	50–90	0.001 (0.000)	0.002 (0.016)	0.002 (0.035)	0.041 (0.117)	0.993	0.982	0.967	0.908
	60–90	0.001 (0.000)	0.001 (0.015)	0.002 (0.034)	0.041 (0.114)	0.993	0.983	0.968	0.909
	70–90	0.001 (0.000)	0.001 (0.014)	0.001 (0.031)	0.037 (0.108)	0.993	0.984	0.971	0.911
	80–90	0.000 (0.000)	0.000 (0.014)	0.002 (0.030)	0.033 (0.092)	1.000	0.985	0.972	0.926
	90–90	0.000 (0.000)	0.000 (0.013)	0.003 (0.027)	0.029 (0.083)	1.000	0.985	0.974	0.933
BP_ND_ IV	10–90	0.003 (0.000)	0.014 (0.035)	0.014 (0.074)	0.085 (0.184)	0.993	0.964	0.943	0.848
	20–90	0.004 (0.000)	0.004 (0.024)	0.003 (0.047)	0.036 (0.148)	0.993	0.975	0.956	0.891
	30–90	0.005 (0.000)	0.001 (0.023)	0.006 (0.046)	0.011 (0.115)	0.993	0.976	0.958	0.904
	40–90	0.001 (0.000)	0.004 (0.023)	0.003 (0.046)	0.008 (0.108)	0.993	0.976	0.958	0.907
	50–90	0.001 (0.000)	0.004 (0.024)	0.002 (0.045)	0.006 (0.107)	0.993	0.975	0.959	0.909
	60–90	0.001 (0.000)	0.005 (0.022)	0.002 (0.043)	0.007 (0.099)	0.993	0.976	0.961	0.917
	70–90	0.001 (0.000)	0.004 (0.022)	0.003 (0.040)	0.006 (0.092)	0.993	0.977	0.964	0.924
	80–90	0.000 (0.000)	0.005 (0.021)	0.000 (0.037)	0.003 (0.089)	1.000	0.977	0.966	0.926
	90–90	0.000 (0.000)	0.005 (0.019)	0.000 (0.034)	0.012 (0.091)	1.000	0.978	0.970	0.925
BP_ND_ V	10–90	0.005 (0.000)	0.005 (0.044)	0.024 (0.122)	0.029 (0.111)	0.993	0.960	0.911	0.903
	20–90	0.006 (0.000)	0.008 (0.027)	0.015 (0.056)	0.008 (0.147)	0.988	0.971	0.950	0.882
	30–90	0.007 (0.000)	0.008 (0.027)	0.021 (0.051)	0.011 (0.165)	0.988	0.972	0.954	0.870
	40–90	0.002 (0.000)	0.002 (0.026)	0.016 (0.052)	0.018 (0.172)	0.993	0.973	0.955	0.868
	50–90	0.002 (0.000)	0.002 (0.026)	0.016 (0.052)	0.010 (0.156)	0.993	0.973	0.955	0.878
	60–90	0.001 (0.000)	0.002 (0.025)	0.016 (0.051)	0.015 (0.156)	0.993	0.974	0.955	0.875
	70–90	0.001 (0.000)	0.002 (0.024)	0.012 (0.050)	0.022 (0.153)	0.993	0.976	0.955	0.876
	80–90	0.000 (0.000)	0.002 (0.022)	0.008 (0.045)	0.027 (0.147)	0.993	0.978	0.959	0.876
	90–90	0.000 (0.000)	0.002 (0.020)	0.005 (0.041)	0.026 (0.135)	0.993	0.979	0.963	0.888

Absolute bias in ${\mathrm{BP}}_{\mathrm{ND}}^{\mathrm{sim}}$ and the area under the curve (AUC) for the acceptability curves across all noise levels (0–5%) for [^18^F]florbetaben

## Discussion

The present pharmacokinetic simulation study demonstrated that, for [^18^F]flutemetamol and [^18^F]florbetaben, the introduction of a break with a maximum of 60 min in a dual-time-window acquisition protocol (early interval of 0–30 min followed by a late interval of 90–110 min) results in a minimal loss in quantitative accuracy while presenting major logistic advantages as compared with full dynamic acquisitions. Therefore, this protocol could serve as suitable alternative in research or clinical trial settings where accurate and fully quantitative measurements might be required.

Analysis of the 2T4k_V_b_ noiseless full TACs showed a systematic bias (0.17–1.95% for [^18^F]flutemetamol and 2.62–6.04% for [^18^F]florbetaben) in SRTM-derived DVR values compared with simulated DVR values. These findings are in line with previous studies reporting that kinetics of [^18^F]flutemetamol and [^18^F]florbetaben are better described by a two-tissue compartment model in target as well as reference tissues [8, 18]. In addition, Nelissen et al. showed that there were similar levels of binding in the second reference tissue compartment for both healthy control and AD subjects and therefore concluded that this binding is likely due to (relatively slow) non-specific retention [18]. As this violates one of the assumptions of SRTM, a slight bias in DVR estimates can be expected [19, 30]. Given the aim of validating a dual-time-window protocol for a reference tissue-based approach, TACs were both generated and fitted according to the SRTM model to prevent a systematic bias in the results.

A first examination of SRTM-derived ${\mathrm{BP}}_{\mathrm{ND}}^{\mathrm{fit}}$ values revealed that most outliers were observed for fits of the 10–90-min interval, and, to a lesser extent, also for fits of the 20–90-min interval. In addition, compared with the full-kinetic curve, the bias in DVR only exceeded previously reported [^11^C]PIB TRT values, of which [^18^F]flutemetamol is an analog, for the 10–90 and 20–90-min intervals for [^18^F]flutemetamol [9, 10]. For the other dual-time-window protocols, the bias remained ≤ 3.1%. Analysis of [^18^F]florbetaben data showed a bias of ≤ 9.1% for the 10–90-min interval and ≤ 5.0% for the 20–90 interval for the highest noise levels, while for all intervals it was ≤ 1.9%. The latter well within previously reported TRT values for [^18^F]florbetaben SUVR data (ranging between 2.9% HC and 6.2% AD) [31]. Reported AUC values also showed a general trend of worse values for longer breaks and higher noise level, with the exception of some extremely low BP_ND_ cases, where the performance of SRTM is known to be suboptimal [10].

Finally, the bias plots of SRTM-generated TACs demonstrated that bias in SRTM-derived *R*_1_ increased as a function of noise and interval for both tracers. More specifically, a larger error in *R*_1_ (> 3% for [^18^F]flutemetamol and > 1% for [^18^F]florbetaben) was observed for the 10–90-min intervals compared with the other intervals. For practical applications, this error would be negligible since the TRT of flow is known to be approximately 9% [32]. As expected, the results showed that the length of the interval is related to bias in ${\mathrm{BP}}_{\mathrm{ND}}^{\mathrm{fit}}$ or DVR and the number of outliers. More specifically, results suggest that it is not advisable to use the 10–90 and 20–90-min intervals for full quantification, especially due to the relatively large percentage of outliers and larger bias in DVR compared with other intervals. Moreover, the observed larger amount of unusable data would result in smaller power to detect changes in clinical trials.

Shorter scan durations are better for the patient and, as such, longer breaks would be preferred. Since the 10–90 and 20–90-min intervals result in a large number of outliers and larger bias, the 30–90-min interval would be a good compromise. This interval would have the additional advantage of a 60-min break, which may allow for interleaved scanning protocols. Consequently, the 30–90-min interval is recommended as the optimal trade-off between patient comfort and quantitative accuracy (bias in DVR < 2% and a maximum of 18% outliers for highest noise level and ${\mathrm{BP}}_{\mathrm{ND}}^{\mathrm{fit}}$). This conclusion is in agreement with recent work of Bullich et al. regarding the optimal [^18^F]florbetaben dual-time-window protocol [17]. Based on their analysis of clinical data, which did not include the 90–110-min diagnostic window, a dual-time-window protocol of 0–30 and 120–140 min was described as optimal. However, their simulations also supported that 0–30 and 90–110-min scanning times would maintain the best compromise between quantitative accuracy and patient comfort. The present simulation study, including TACs representing the AD spectrum and different noise levels, further validated their findings.

A major advantage of a 60-min gap in the scanning protocol is that it allows for interleaved scanning protocols, in which the first scan of the second patient can be acquired within the resting period (interval) of the first patient. An interleaved scanning protocol would increase both patient throughput and efficient use of tracer batches, thereby decreasing costs. An assessment of the practical feasibility of such an interleaved scanning protocol is beyond the scope of the present study and needs to be addressed in future studies. Main limitations of the current study include the use of fixed *K*_1_ and *k*_2_ parameters for simulations, the limited sample size of the available clinical dataset, and the extrapolation of TRT variability from other radiotracers to this work. The first limits the possibility of assessing the impact of changes in cerebral blood flow on dual-time-window protocol-based quantification, but it can be expected to introduce only small additional bias over and above the one introduced by the protocol itself [11]. Regarding the second, additional clinical data would have allowed the verification of the simulation results, which remains a goal for future work once larger cohorts are available. With respect to extrapolating TRT variability, although values from other tracers might not directly translate to our data, they are expected to be in comparable ranges [24]. In addition, although outside of the scope of this study, the evaluation of parametric methods for quantification of dual-time-window-derived data is warranted, which would require imaging data in order to optimize image contrast and reduce noise and artifacts. Finally, it must be noted that the goal of this study was not to compare these two tracers, but to identify the optimal dynamic dual-time-window scanning protocol for both of them. In order to make a head-to-head comparison between tracers, PET imaging data from both tracers within the same patient would be required.

## Conclusion

Accurate estimates of ${\mathrm{BP}}_{\mathrm{ND}}^{\mathrm{fit}}$ can be obtained for both [^18^F]flutemetamol and [^18^F]florbetaben using a 60-min dual-time-window protocol, with dynamic scanning from 0 to 30 and again from 90 to 110 min. This protocol results in a limited number of outliers, and an acceptable bias in ${\mathrm{BP}}_{\mathrm{ND}}^{\mathrm{fit}}$ and DVR estimates. Moreover, it enables interleaved scanning protocols, optimizing tracer batch usage and patient throughput, thereby reducing costs and improving patient comfort.

## Additional file

Additional file 1: **Figure S2.** Interpolation of two different intervals in a reference tissue TAC for [^18^F]florbetaben. **Table S1a.** Boundary values of [^18^F]flutemetamol kinetic parameters. **Table S1b.** Boundary values of [^18^F]florbetaben kinetic parameters. **Table S2.** Bias in DVR and outliers as a result of noise and the dual-time-window protocol for SRTM-generated [^18^F]flutemetamol TACs. **Table S3.** Bias in DVR and outliers as a result of noise and the dual-time-window protocol for SRTM generated [^18^F]florbetaben TACs. **Table S4.** Absolute bias in BP_ND_ and AUC as a result of noise and the dual-time-window protocol for SRTM generated [^18^F]flutemetamol TACs. **Table S5.** Absolute bias in BP_ND_ and AUC as a result of noise and the dual-time-window protocol for SRTM generated [^18^F]florbetaben TACs. (DOCX 137 kb)

## Abbreviations

[^11^C]PiB: Carbon-11 Pittsburgh Compound B
18F: Fluorine-18
2T4k_V_b_: Reversible two tissue compartment model (4 rate constants) with additional blood volume fraction parameter
AD: Alzheimer’s disease
AUC: Area under the curve
Aβ: Amyloid-beta
BP_ND_: Non-displaceable binding potential
COV: Coefficient of variation
DVR: Distribution volume ratio
FDG: Fluorodeoxyglucose
FRTM: Full reference tissue model
MRTM: Multilinear reference tissue method
PET: Positron emission tomography
RPM: Receptor parametric mapping
SRTM: Simplified reference tissue method
SUVR: Standardized uptake value ratio
TAC: Time activity curve
TRT: Test-retest
VOI: Volume of interest
