# Supplementary material for: Optimized dual-time-window protocols for quantitative [18F]flutemetamol and [18F]florbetaben PET studies
Source: EJNMMI Res. 2019 Mar 27;9:32. doi: 10.1186/s13550-019-0499-4 (PMC6437225; doi:10.1186/s13550-019-0499-4)
Supplement: Supplementary file 1 — Figure S2. Interpolation of two different intervals in a reference tissue TAC for [18F]florbetaben. Table S1a. Boundary values of [18F]flutemetamol kinetic parameters. Table S1b. Boundary values of [18F]florbetaben kinetic parameters. Table S2. Bias in DVR and outliers as a result of noise and the dual-time-window protocol for SRTM-generated [18F]flutemetamol TACs. Table S3. Bias in DVR and outliers as a result of noise and the dual-time-window protocol for SRTM generated [18F]florbetaben TACs. Table S4. Absolute bias in BPND and AUC as a result of noise and the dual-time-window protocol for SRTM generated [18F]flutemetamol TACs. Table S5. Absolute bias in BPND and AUC as a result of noise and the dual-time-window protocol for SRTM generated [18F]florbetaben TACs. (DOCX 137 kb) [file 13550_2019_499_MOESM1_ESM.docx]

**Supplementary Figure 2.** Interpolation of two different intervals in a reference tissue TAC for [^18^F]florbetaben.

**Supplementary Figure 1.** Interpolation of two different intervals in a reference tissue TAC for [^18^F]flutemetamol.

**Supplementary Table 1a.** Boundary values of [^18^F]flutemetamol kinetic parameters

|  | ***K*_1_** | ***k*_2_** | ***k*_3_** | ***k*_3_/*k*_4_** | **Vt** | **V_b_** |
| --- | --- | --- | --- | --- | --- | --- |
|  | min max | min max | min max | min max | min max | min max |
| **2T4k_V_b_** | 0 1 |  | 0.005 1 | 0 20 | 0 50 | 0 0.5 |
|  | | | | | | |
| **FRTM** | 0 100 | 0.005 1 | 0.001 1 | 0 20 |  |  |
|  | | | | | | |
| **SRTM** | 0 100 | 0.005 1 |  | 0 20 |  |  |

**Supplementary Table 1b.** Boundary values of [^18^F]florbetaben kinetic parameters

|  | ***K*_1_** | ***k*_2_** | ***k*_3_** | ***k*_3_/*k*_4_** | **Vt** | **V_b_** |
| --- | --- | --- | --- | --- | --- | --- |
|  | min max | min max | min max | min max | min max | min max |
| **2T4k_V_b_** | 0 1 |  | 0.001 1 | 0 20 | 0 50 | 0 0.5 |
|  | | | | | | |
| **FRTM** | 0 100 | 0.005 1 | 0.001 1 | 0 20 |  |  |
|  | | | | | | |
| **SRTM** | 0 100 | 0.005 1 |  | 0 20 |  |  |

| **Supplementary Table 2.** Bias in DVR and outliers as a result of noise and the dual-time-window protocol for SRTM generated [^18^F]flutemetamol TACs | | | | | | | | |
| --- | --- | --- | --- | --- | --- | --- | --- | --- |
| FLUT |  | % Bias DVR | | |  | % Outlier | | |
|  | Interval | 1% | 2% | 5% |  | 1% | 2% | 5% |
| *BP*_ND_ I | 10-90 | 0.0 | 1.9 | 2.0 |  | 0.0 | 0.0 | 0.0 |
|  | 20-90 | 6.4 | 6.1 | 2.4 |  | 0.0 | 0.0 | 0.0 |
|  | 30-90 | 0.5 | 0.5 | 3.1 |  | 0.0 | 0.0 | 0.0 |
|  | 40-90 | 1.5 | 1.6 | 1.6 |  | 0.0 | 0.0 | 0.0 |
|  | 50-90 | 1.5 | 1.5 | 1.7 |  | 0.0 | 0.0 | 0.0 |
|  | 60-90 | 1.5 | 1.6 | 1.9 |  | 0.0 | 0.0 | 0.0 |
|  | 70-90 | 1.8 | 2.1 | 0.3 |  | 0.0 | 0.0 | 0.0 |
|  | 80-90 | 0.8 | 1.3 | 1.3 |  | 0.0 | 0.0 | 0.0 |
|  | 90-90 | 0.0 | 0.0 | 0.0 |  | 0.0 | 0.0 | 0.0 |
|  |  |  |  |  |  |  |  |  |
| *BP*_ND_ II | 10-90 | 1.0 | 6.2 | 8.2 |  | 0.0 | 2.0 | 12.0 |
|  | 20-90 | 1.5 | 0.2 | 4.0 |  | 0.0 | 0.0 | 2.0 |
|  | 30-90 | 0.1 | 0.2 | 1.3 |  | 0.0 | 0.0 | 0.0 |
|  | 40-90 | 0.0 | 0.3 | 0.1 |  | 0.0 | 0.0 | 0.0 |
|  | 50-90 | 0.0 | 0.3 | 0.4 |  | 0.0 | 0.0 | 0.0 |
|  | 60-90 | 0.0 | 0.2 | 0.6 |  | 0.0 | 0.0 | 0.0 |
|  | 70-90 | 0.0 | 0.1 | 0.6 |  | 0.0 | 0.0 | 0.0 |
|  | 80-90 | 0.1 | 0.0 | 0.3 |  | 0.0 | 0.0 | 0.0 |
|  | 90-90 | 0.0 | 0.0 | 0.0 |  | 0.0 | 0.0 | 0.0 |
|  |  |  |  |  |  |  |  |  |
| *BP*_ND_ III | 10-90 | 0.9 | 1.4 | 2.0 |  | 0.0 | 10.0 | 34.0 |
|  | 20-90 | 0.1 | 0.5 | 0.6 |  | 0.0 | 0.0 | 10.0 |
|  | 30-90 | 0.1 | 0.3 | 0.3 |  | 0.0 | 0.0 | 4.0 |
|  | 40-90 | 0.1 | 0.5 | 0.0 |  | 0.0 | 0.0 | 4.0 |
|  | 50-90 | 0.1 | 0.5 | 0.2 |  | 0.0 | 0.0 | 4.0 |
|  | 60-90 | 0.1 | 0.5 | 0.1 |  | 0.0 | 0.0 | 4.0 |
|  | 70-90 | 0.1 | 0.4 | 0.3 |  | 0.0 | 0.0 | 4.0 |
|  | 80-90 | 0.0 | 0.1 | 0.5 |  | 0.0 | 0.0 | 2.0 |
|  | 90-90 | 0.0 | 0.0 | 0.0 |  | 0.0 | 0.0 | 2.0 |
|  |  |  |  |  |  |  |  |  |
| *BP*_ND_ IV | 10-90 | 1.6 | 2.7 | 1.8 |  | 0.0 | 2.0 | 32.0 |
|  | 20-90 | 0.2 | 0.3 | 0.8 |  | 0.0 | 0.0 | 12.0 |
|  | 30-90 | 0.0 | 0.0 | 1.3 |  | 0.0 | 0.0 | 6.0 |
|  | 40-90 | 0.0 | 0.1 | 0.6 |  | 0.0 | 0.0 | 0.0 |
|  | 50-90 | 0.0 | 0.1 | 0.7 |  | 0.0 | 0.0 | 0.0 |
|  | 60-90 | 0.0 | 0.1 | 0.7 |  | 0.0 | 0.0 | 0.0 |
|  | 70-90 | 0.1 | 0.2 | 0.7 |  | 0.0 | 0.0 | 0.0 |
|  | 80-90 | 0.0 | 0.1 | 0.9 |  | 0.0 | 0.0 | 0.0 |
|  | 90-90 | 0.0 | 0.0 | 0.0 |  | 0.0 | 0.0 | 0.0 |
|  |  |  |  |  |  |  |  |  |
| *BP*_ND_ V | 10-90 | 1.2 | 0.1 | 6.5 |  | 2.0 | 14.0 | 48.0 |
|  | 20-90 | 0.1 | 0.0 | 0.5 |  | 0.0 | 0.0 | 22.0 |
|  | 30-90 | 0.0 | 0.6 | 0.8 |  | 0.0 | 0.0 | 18.0 |
|  | 40-90 | 0.0 | 0.5 | 1.0 |  | 0.0 | 0.0 | 16.0 |
|  | 50-90 | 0.0 | 0.6 | 0.9 |  | 0.0 | 0.0 | 14.0 |
|  | 60-90 | 0.0 | 0.6 | 0.3 |  | 0.0 | 0.0 | 12.0 |
|  | 70-90 | 0.0 | 0.4 | 0.5 |  | 0.0 | 0.0 | 14.0 |
|  | 80-90 | 0.0 | 0.2 | 0.4 |  | 0.0 | 0.0 | 12.0 |
|  | 90-90 | 0.0 | 0.0 | 0.0 |  | 0.0 | 0.0 | 12.0 |

Bias in simulated DVR compared to full-kinetic curve DVR and % outliers across

noise levels (1-5%) for [^18^F]flutemetamol.

| **Supplementary Table 3.** Bias in DVR and outliers as a result of noise and the dual-time-window protocol for SRTM generated [^18^F]florbetaben TACs | | | | | | | | |
| --- | --- | --- | --- | --- | --- | --- | --- | --- |
| FLUT |  | % Bias DVR | | |  | % Outlier | | |
|  | Interval | 1% | 2% | 5% |  | 1% | 2% | 5% |
| *BP*_ND_ I | 10 90 | 2.1 | 0.9 | 3.3 |  | 0.0 | 0.0 | 0.0 |
|  | 20 90 | 0.9 | 0.2 | 2.3 |  | 0.0 | 0.0 | 0.0 |
|  | 30 90 | 1.0 | 1.2 | 1.8 |  | 0.0 | 0.0 | 0.0 |
|  | 40 90 | 0.6 | 1.9 | 0.3 |  | 0.0 | 0.0 | 0.0 |
|  | 50 90 | 0.1 | 1.1 | 1.2 |  | 0.0 | 0.0 | 0.0 |
|  | 60 90 | 1.0 | 1.0 | 0.4 |  | 0.0 | 0.0 | 0.0 |
|  | 70 90 | 1.0 | 1.1 | 0.7 |  | 0.0 | 0.0 | 0.0 |
|  | 80 90 | 0.5 | 0.4 | 0.4 |  | 0.0 | 0.0 | 0.0 |
|  | 90 90 | 0.0 | 0.0 | 0.0 |  | 0.0 | 0.0 | 0.0 |
|  |  |  |  |  |  |  |  |  |
| *BP*_ND_ II | 10 90 | 0.1 | 3.4 | 9.1 |  | 0.0 | 0.0 | 0.0 |
|  | 20 90 | 0.1 | 0.1 | 5.0 |  | 0.0 | 0.0 | 0.0 |
|  | 30 90 | 0.1 | 0.4 | 0.2 |  | 0.0 | 0.0 | 0.0 |
|  | 40 90 | 0.0 | 0.2 | 0.1 |  | 0.0 | 0.0 | 0.0 |
|  | 50 90 | 0.0 | 0.2 | 0.3 |  | 0.0 | 0.0 | 0.0 |
|  | 60 90 | 0.1 | 0.1 | 0.3 |  | 0.0 | 0.0 | 0.0 |
|  | 70 90 | 0.0 | 0.1 | 0.3 |  | 0.0 | 0.0 | 0.0 |
|  | 80 90 | 0.0 | 0.0 | 0.2 |  | 0.0 | 0.0 | 0.0 |
|  | 90 90 | 0.0 | 0.0 | 0.0 |  | 0.0 | 0.0 | 0.0 |
|  |  |  |  |  |  |  |  |  |
| *BP*_ND_ III | 10 90 | 0.2 | 0.7 | 3.5 |  | 0.0 | 2.0 | 16.0 |
|  | 20 90 | 0.2 | 0.2 | 1.8 |  | 0.0 | 0.0 | 0.0 |
|  | 30 90 | 0.3 | 0.5 | 0.6 |  | 0.0 | 0.0 | 0.0 |
|  | 40 90 | 0.2 | 0.4 | 0.7 |  | 0.0 | 0.0 | 0.0 |
|  | 50 90 | 0.1 | 0.4 | 0.7 |  | 0.0 | 0.0 | 0.0 |
|  | 60 90 | 0.1 | 0.4 | 0.8 |  | 0.0 | 0.0 | 0.0 |
|  | 70 90 | 0.0 | 0.3 | 0.5 |  | 0.0 | 0.0 | 0.0 |
|  | 80 90 | 0.0 | 0.1 | 0.3 |  | 0.0 | 0.0 | 0.0 |
|  | 90 90 | 0.0 | 0.0 | 0.0 |  | 0.0 | 0.0 | 0.0 |
|  |  |  |  |  |  |  |  |  |
| *BP*_ND_ IV | 10 90 | 0.5 | 0.8 | 4.0 |  | 0.0 | 0.0 | 20.0 |
|  | 20 90 | 0.0 | 0.2 | 1.3 |  | 0.0 | 0.0 | 0.0 |
|  | 30 90 | 0.2 | 0.3 | 0.1 |  | 0.0 | 0.0 | 0.0 |
|  | 40 90 | 0.0 | 0.2 | 0.2 |  | 0.0 | 0.0 | 0.0 |
|  | 50 90 | 0.0 | 0.1 | 0.3 |  | 0.0 | 0.0 | 0.0 |
|  | 60 90 | 0.0 | 0.1 | 0.3 |  | 0.0 | 0.0 | 0.0 |
|  | 70 90 | 0.1 | 0.1 | 0.3 |  | 0.0 | 0.0 | 0.0 |
|  | 80 90 | 0.0 | 0.0 | 0.5 |  | 0.0 | 0.0 | 0.0 |
|  | 90 90 | 0.0 | 0.0 | 0.0 |  | 0.0 | 0.0 | 0.0 |
|  |  |  |  |  |  |  |  |  |
| *BP*_ND_ V | 10 90 | 0.4 | 1.4 | 2.7 |  | 0.0 | 0.0 | 32.0 |
|  | 20 90 | 0.3 | 0.5 | 0.9 |  | 0.0 | 0.0 | 10.0 |
|  | 30 90 | 0.3 | 0.8 | 0.7 |  | 0.0 | 0.0 | 2.0 |
|  | 40 90 | 0.0 | 0.6 | 0.4 |  | 0.0 | 0.0 | 0.0 |
|  | 50 90 | 0.0 | 0.6 | 0.8 |  | 0.0 | 0.0 | 2.0 |
|  | 60 90 | 0.0 | 0.6 | 0.6 |  | 0.0 | 0.0 | 2.0 |
|  | 70 90 | 0.0 | 0.4 | 0.2 |  | 0.0 | 0.0 | 2.0 |
|  | 80 90 | 0.0 | 0.2 | 0.0 |  | 0.0 | 0.0 | 2.0 |
|  | 90 90 | 0.0 | 0.0 | 0.0 |  | 0.0 | 0.0 | 0.0 |

Bias in simulated DVR compared to full-kinetic curve DVR and % outliers

across noise levels (1-5%) for [^18^F]florbetaben.

| **Supplementary Table 4.** Absolute bias in *BP*_ND_ and AUC as a result of noise and the dual-time-window protocol for SRTM generated [^18^F]flutemetamol TACs | | | | | | | | | | |
| --- | --- | --- | --- | --- | --- | --- | --- | --- | --- | --- |
| FLUT |  | Absolute mean bias *BP*_ND_ (SD) | | | |  | AUC | | | |
|  | Interval | 0% | 1% | 2% | 5% |  | 0% | 1% | 2% | 5% |
| *BP*_ND_ I | 10-90 | 0.001 (0.000) | 0.017 (0.058) | 0.042 (0.079) | 0.093 (0.154) |  | 0.993 | 0.971 | 0.943 | 0.894 |
|  | 20-90 | 0.001 (0.000) | 0.084 (0.283) | 0.086 (0.276) | 0.098 (0.211) |  | 0.993 | 0.908 | 0.902 | 0.887 |
|  | 30-90 | 0.001 (0.000) | 0.012 (0.079) | 0.017 (0.074) | 0.105 (0.222) |  | 0.993 | 0.969 | 0.961 | 0.878 |
|  | 40-90 | 0.001 (0.000) | 0.002 (0.011) | 0.006 (0.022) | 0.055 (0.113) |  | 0.993 | 0.986 | 0.977 | 0.929 |
|  | 50-90 | 0.000 (0.000) | 0.002 (0.011) | 0.007 (0.025) | 0.053 (0.116) |  | 0.993 | 0.986 | 0.976 | 0.929 |
|  | 60-90 | 0.000 (0.000) | 0.002 (0.029) | 0.006 (0.045) | 0.051 (0.115) |  | 0.993 | 0.981 | 0.974 | 0.932 |
|  | 70-90 | 0.000 (0.000) | 0.036 (0.119) | 0.000 (0.020) | 0.075 (0.147) |  | 0.993 | 0.948 | 0.978 | 0.909 |
|  | 80-90 | 0.000 (0.000) | 0.026 (0.174) | 0.035 (0.181) | 0.058 (0.133) |  | 1.000 | 0.955 | 0.946 | 0.923 |
|  | 90-90 | 0.000 (0.000) | 0.018 (0.162) | 0.022 (0.162) | 0.072 (0.202) |  | 1.000 | 0.962 | 0.958 | 0.91 |
|  |  |  | | |  |  |  |  |  |  |
| *BP*_ND_ II | 10-90 | 0.002 (0.000) | 0.014 (0.046) | 0.081 (0.217) | 0.128 (0.271) |  | 0.993 | 0.97 | 0.895 | 0.838 |
|  | 20-90 | 0.001 (0.000) | 0.021 (0.135) | 0.009 (0.040) | 0.076 (0.200) |  | 0.993 | 0.963 | 0.965 | 0.881 |
|  | 30-90 | 0.001 (0.000) | 0.002 (0.015) | 0.003 (0.030) | 0.043 (0.159) |  | 0.993 | 0.982 | 0.971 | 0.906 |
|  | 40-90 | 0.000 (0.000) | 0.002 (0.015) | 0.003 (0.029) | 0.029 (0.112) |  | 0.993 | 0.982 | 0.971 | 0.919 |
|  | 50-90 | 0.000 (0.000) | 0.002 (0.015) | 0.002 (0.029) | 0.022 (0.097) |  | 0.993 | 0.982 | 0.972 | 0.923 |
|  | 60-90 | 0.000 (0.000) | 0.003 (0.014) | 0.004 (0.029) | 0.020 (0.095) |  | 0.993 | 0.983 | 0.972 | 0.922 |
|  | 70-90 | 0.000 (0.000) | 0.002 (0.014) | 0.005 (0.029) | 0.020 (0.096) |  | 0.993 | 0.984 | 0.972 | 0.924 |
|  | 80-90 | 0.000 (0.000) | 0.002 (0.013) | 0.007 (0.027) | 0.023 (0.098) |  | 1.000 | 0.985 | 0.973 | 0.924 |
|  | 90-90 | 0.000 (0.000) | 0.003 (0.013) | 0.006 (0.024) | 0.027 (0.098) |  | 1.000 | 0.985 | 0.975 | 0.925 |
|  |  |  | | | |  |  |  |  |  |
| *BP*_ND_ III | 10-90 | 0.003 (0.000) | 0.014 (0.063) | 0.031 (0.099) | 0.023 (0.128) |  | 0.993 | 0.956 | 0.924 | 0.898 |
|  | 20-90 | 0.002 (0.000) | 0.001 (0.026) | 0.019 (0.080) | 0.06 (0.145) |  | 0.993 | 0.975 | 0.946 | 0.876 |
|  | 30-90 | 0.001 (0.000) | 0.001 (0.023) | 0.007 (0.054) | 0.056 (0.158) |  | 0.993 | 0.977 | 0.956 | 0.881 |
|  | 40-90 | 0.001 (0.000) | 0.001 (0.023) | 0.004 (0.049) | 0.051 (0.146) |  | 0.993 | 0.978 | 0.958 | 0.885 |
|  | 50-90 | 0.001 (0.000) | 0.001 (0.023) | 0.005 (0.048) | 0.049 (0.147) |  | 0.993 | 0.978 | 0.959 | 0.886 |
|  | 60-90 | 0.001 (0.000) | 0.001 (0.022) | 0.005 (0.048) | 0.05 (0.147) |  | 0.993 | 0.978 | 0.959 | 0.887 |
|  | 70-90 | 0.001 (0.000) | 0.000 (0.021) | 0.006 (0.047) | 0.047 (0.144) |  | 0.993 | 0.979 | 0.96 | 0.888 |
|  | 80-90 | 0.000 (0.000) | 0.001 (0.022) | 0.01 (0.046) | 0.059 (0.164) |  | 1.000 | 0.979 | 0.961 | 0.883 |
|  | 90-90 | 0.000 (0.000) | 0.001 (0.021) | 0.012 (0.043) | 0.051 (0.142) |  | 1.000 | 0.979 | 0.963 | 0.894 |
|  |  |  | | |  |  |  |  |  |  |
| *BP*_ND_ IV | 10-90 | 0.005 (0.000) | 0.034 (0.082) | 0.041 (0.147) | 0.027 (0.192) |  | 0.993 | 0.932 | 0.892 | 0.854 |
|  | 20-90 | 0.003 (0.000) | 0.012 (0.038) | 0.004 (0.067) | 0.043 (0.183) |  | 0.993 | 0.963 | 0.942 | 0.859 |
|  | 30-90 | 0.002 (0.000) | 0.008 (0.032) | 0.002 (0.055) | 0.036 (0.168) |  | 0.993 | 0.968 | 0.949 | 0.866 |
|  | 40-90 | 0.001 (0.000) | 0.008 (0.030) | 0.003 (0.054) | 0.048 (0.173) |  | 0.993 | 0.97 | 0.95 | 0.858 |
|  | 50-90 | 0.001 (0.000) | 0.007 (0.031) | 0.003 (0.054) | 0.045 (0.174) |  | 0.993 | 0.97 | 0.949 | 0.858 |
|  | 60-90 | 0.001 (0.000) | 0.008 (0.030) | 0.003 (0.053) | 0.046 (0.169) |  | 0.993 | 0.969 | 0.951 | 0.863 |
|  | 70-90 | 0.001 (0.000) | 0.007 (0.031) | 0.004 (0.051) | 0.045 (0.165) |  | 0.993 | 0.969 | 0.953 | 0.868 |
|  | 80-90 | 0.000 (0.000) | 0.008 (0.030) | 0.000 (0.050) | 0.043 (0.163) |  | 1.000 | 0.969 | 0.954 | 0.867 |
|  | 90-90 | 0.000 (0.000) | 0.008 (0.028) | 0.001 (0.045) | 0.057 (0.179) |  | 1.000 | 0.97 | 0.959 | 0.861 |
|  |  |  | | | |  |  |  |  |  |
| *BP*_ND_ V | 10-90 | 0.006 (0.000) | 0.023 (0.082) | 0.005 (0.145) | 0.117 (0.111) |  | 0.988 | 0.929 | 0.885 | 0.852 |
|  | 20-90 | 0.004 (0.000) | 0.001 (0.042) | 0.004 (0.090) | 0.011 (0.179) |  | 0.993 | 0.96 | 0.925 | 0.85 |
|  | 30-90 | 0.002 (0.000) | 0.000 (0.037) | 0.014 (0.074) | 0.015 (0.179) |  | 0.993 | 0.965 | 0.938 | 0.851 |
|  | 40-90 | 0.001 (0.000) | 0.001 (0.034) | 0.014 (0.072) | 0.019 (0.168) |  | 0.993 | 0.968 | 0.94 | 0.853 |
|  | 50-90 | 0.001 (0.000) | 0.001 (0.034) | 0.015 (0.071) | 0.018 (0.164) |  | 0.993 | 0.968 | 0.94 | 0.855 |
|  | 60-90 | 0.001 (0.000) | 0.001 (0.034) | 0.015 (0.071) | 0.008 (0.173) |  | 0.993 | 0.968 | 0.94 | 0.849 |
|  | 70-90 | 0.001 (0.000) | 0.001 (0.033) | 0.012 (0.072) | 0.01 (0.163) |  | 0.993 | 0.968 | 0.939 | 0.856 |
|  | 80-90 | 0.000 (0.000) | 0.001 (0.031) | 0.008 (0.067) | 0.006 (0.172) |  | 1.000 | 0.969 | 0.943 | 0.849 |
|  | 90-90 | 0.000 (0.000) | 0.001 (0.029) | 0.004 (0.061) | 0.002 (0.150) |  | 1.000 | 0.971 | 0.949 | 0.867 |

Absolute bias in ${BP}_{ND}^{sim}$ and the area under the curve (AUC) for the acceptability curves across all

noise levels (0-5%) for [^18^F]flutemetamol.

| **Supplementary Table 5.** Absolute bias in *BP*_ND_ and AUC as a result of noise and the dual-time-window protocol for SRTM generated [^18^F]florbetaben TACs | | | | | | | | | | |
| --- | --- | --- | --- | --- | --- | --- | --- | --- | --- | --- |
| FBB |  | Absolute mean bias *BP*_ND_ (SD) | | | |  | AUC | | | |
|  | Interval | 0% | 1% | 2% | 5% |  | 0% | 1% | 2% | 5% |
| *BP*_ND_ I | 10-90 | 0.000 (0.000) | 0.012 (0.026) | 0.021 (0.043) | 0.059 (0.080) |  | 0.993 | 0.979 | 0.966 | 0.929 |
|  | 20-90 | 0.000 (0.000) | 0.000 (0.011) | 0.013 (0.040) | 0.048 (0.076) |  | 0.993 | 0.986 | 0.971 | 0.937 |
|  | 30-90 | 0.000 (0.000) | 0.000 (0.008) | 0.001 (0.028) | 0.006 (0.046) |  | 0.993 | 0.988 | 0.979 | 0.968 |
|  | 40-90 | 0.000 (0.000) | 0.003 (0.032) | 0.008 (0.014) | 0.021 (0.063) |  | 0.993 | 0.978 | 0.981 | 0.956 |
|  | 50-90 | 0.000 (0.000) | 0.009 (0.012) | 0.000 (0.037) | 0.012 (0.056) |  | 0.993 | 0.983 | 0.978 | 0.962 |
|  | 60-90 | 0.000 (0.000) | 0.000 (0.007) | 0.001 (0.015) | 0.029 (0.063) |  | 0.993 | 0.989 | 0.983 | 0.956 |
|  | 70-90 | 0.000 (0.000) | 0.001 (0.006) | 0.000 (0.013) | 0.032 (0.069) |  | 0.993 | 0.99 | 0.984 | 0.953 |
|  | 80-90 | 0.000 (0.000) | 0.005 (0.036) | 0.007 (0.066) | 0.029 (0.081) |  | 1.000 | 0.978 | 0.963 | 0.948 |
|  | 90-90 | 0.000 (0.000) | 0.01 (0.011) | 0.011 (0.064) | 0.024 (0.067) |  | 1.000 | 0.983 | 0.962 | 0.956 |
|  |  |  | | | |  |  |  |  |  |
| *BP*_ND_ II | 10-90 | 0.001 (0.000) | 0.003 (0.013) | 0.045 (0.167) | 0.131 (0.307) |  | 0.993 | 0.984 | 0.935 | 0.861 |
|  | 20-90 | 0.001 (0.000) | 0.000 (0.012) | 0.000 (0.023) | 0.079 (0.207) |  | 0.993 | 0.985 | 0.976 | 0.891 |
|  | 30-90 | 0.002 (0.000) | 0.000 (0.012) | 0.003 (0.023) | 0.016 (0.063) |  | 0.993 | 0.985 | 0.975 | 0.946 |
|  | 40-90 | 0.000 (0.000) | 0.001 (0.012) | 0.001 (0.023) | 0.012 (0.062) |  | 0.993 | 0.985 | 0.976 | 0.946 |
|  | 50-90 | 0.000 (0.000) | 0.001 (0.011) | 0.001 (0.022) | 0.01 (0.060) |  | 0.993 | 0.986 | 0.977 | 0.948 |
|  | 60-90 | 0.000 (0.000) | 0.002 (0.010) | 0.000 (0.021) | 0.009 (0.056) |  | 0.993 | 0.987 | 0.978 | 0.948 |
|  | 70-90 | 0.000 (0.000) | 0.001 (0.009) | 0.001 (0.020) | 0.01 (0.055) |  | 0.993 | 0.988 | 0.979 | 0.95 |
|  | 80-90 | 0.000 (0.000) | 0.001 (0.007) | 0.002 (0.017) | 0.011 (0.053) |  | 1.000 | 0.989 | 0.981 | 0.954 |
|  | 90-90 | 0.000 (0.000) | 0.001 (0.008) | 0.002 (0.015) | 0.014 (0.053) |  | 1.000 | 0.989 | 0.983 | 0.954 |
|  |  |  | | | |  |  |  |  |  |
| *BP*_ND_ III | 10-90 | 0.002 (0.000) | 0.002 (0.022) | 0.014 (0.066) | 0.084 (0.159) |  | 0.993 | 0.978 | 0.955 | 0.875 |
|  | 20-90 | 0.002 (0.000) | 0.003 (0.016) | 0.000 (0.041) | 0.058 (0.117) |  | 0.993 | 0.982 | 0.964 | 0.898 |
|  | 30-90 | 0.003 (0.000) | 0.004 (0.016) | 0.004 (0.035) | 0.038 (0.112) |  | 0.993 | 0.982 | 0.966 | 0.91 |
|  | 40-90 | 0.001 (0.000) | 0.002 (0.016) | 0.003 (0.036) | 0.041 (0.116) |  | 0.993 | 0.982 | 0.966 | 0.909 |
|  | 50-90 | 0.001 (0.000) | 0.002 (0.016) | 0.002 (0.035) | 0.041 (0.117) |  | 0.993 | 0.982 | 0.967 | 0.908 |
|  | 60-90 | 0.001 (0.000) | 0.001 (0.015) | 0.002 (0.034) | 0.041 (0.114) |  | 0.993 | 0.983 | 0.968 | 0.909 |
|  | 70-90 | 0.001 (0.000) | 0.001 (0.014) | 0.001 (0.031) | 0.037 (0.108) |  | 0.993 | 0.984 | 0.971 | 0.911 |
|  | 80-90 | 0.000 (0.000) | 0.000 (0.014) | 0.002 (0.030) | 0.033 (0.092) |  | 1.000 | 0.985 | 0.972 | 0.926 |
|  | 90-90 | 0.000 (0.000) | 0.000 (0.013) | 0.003 (0.027) | 0.029 (0.083) |  | 1.000 | 0.985 | 0.974 | 0.933 |
|  |  |  |  | | |  |  |  |  |  |
| *BP*_ND_ IV | 10-90 | 0.003 (0.000) | 0.014 (0.035) | 0.014 (0.074) | 0.085 (0.184) |  | 0.993 | 0.964 | 0.943 | 0.848 |
|  | 20-90 | 0.004 (0.000) | 0.004 (0.024) | 0.003 (0.047) | 0.036 (0.148) |  | 0.993 | 0.975 | 0.956 | 0.891 |
|  | 30-90 | 0.005 (0.000) | 0.001 (0.023) | 0.006 (0.046) | 0.011 (0.115) |  | 0.993 | 0.976 | 0.958 | 0.904 |
|  | 40-90 | 0.001 (0.000) | 0.004 (0.023) | 0.003 (0.046) | 0.008 (0.108) |  | 0.993 | 0.976 | 0.958 | 0.907 |
|  | 50-90 | 0.001 (0.000) | 0.004 (0.024) | 0.002 (0.045) | 0.006 (0.107) |  | 0.993 | 0.975 | 0.959 | 0.909 |
|  | 60-90 | 0.001 (0.000) | 0.005 (0.022) | 0.002 (0.043) | 0.007 (0.099) |  | 0.993 | 0.976 | 0.961 | 0.917 |
|  | 70-90 | 0.001 (0.000) | 0.004 (0.022) | 0.003 (0.040) | 0.006 (0.092) |  | 0.993 | 0.977 | 0.964 | 0.924 |
|  | 80-90 | 0.000 (0.000) | 0.005 (0.021) | 0.000 (0.037) | 0.003 (0.089) |  | 1.000 | 0.977 | 0.966 | 0.926 |
|  | 90-90 | 0.000 (0.000) | 0.005 (0.019) | 0.000 (0.034) | 0.012 (0.091) |  | 1.000 | 0.978 | 0.97 | 0.925 |
|  |  |  | | | |  |  |  |  |  |
| *BP*_ND_ V | 10-90 | 0.005 (0.000) | 0.005 (0.044) | 0.024 (0.122) | 0.029 (0.111) |  | 0.993 | 0.96 | 0.911 | 0.903 |
|  | 20-90 | 0.006 (0.000) | 0.008 (0.027) | 0.015 (0.056) | 0.008 (0.147) |  | 0.988 | 0.971 | 0.95 | 0.882 |
|  | 30-90 | 0.007 (0.000) | 0.008 (0.027) | 0.021 (0.051) | 0.011 (0.165) |  | 0.988 | 0.972 | 0.954 | 0.87 |
|  | 40-90 | 0.002 (0.000) | 0.002 (0.026) | 0.016 (0.052) | 0.018 (0.172) |  | 0.993 | 0.973 | 0.955 | 0.868 |
|  | 50-90 | 0.002 (0.000) | 0.002 (0.026) | 0.016 (0.052) | 0.010 (0.156) |  | 0.993 | 0.973 | 0.955 | 0.878 |
|  | 60-90 | 0.001 (0.000) | 0.002 (0.025) | 0.016 (0.051) | 0.015 (0.156) |  | 0.993 | 0.974 | 0.955 | 0.875 |
|  | 70-90 | 0.001 (0.000) | 0.002 (0.024) | 0.012 (0.050) | 0.022 (0.153) |  | 0.993 | 0.976 | 0.955 | 0.876 |
|  | 80-90 | 0.000 (0.000) | 0.002 (0.022) | 0.008 (0.045) | 0.027 (0.147) |  | 0.993 | 0.978 | 0.959 | 0.876 |
|  | 90-90 | 0.000 (0.000) | 0.002 (0.020) | 0.005 (0.041) | 0.026 (0.135) |  | 0.993 | 0.979 | 0.963 | 0.888 |

Absolute bias in ${BP}_{ND}^{sim}$ and the area under the curve (AUC) for the acceptability curves across

all noise levels (0-5%) for [^18^F]florbetaben.
